# Cardiac arrest is a predictor of difficult tracheal intubation independent of operator experience in hospitalized patients

**DOI:** 10.1186/1471-2253-14-38

**Published:** 2014-05-22

**Authors:** Nita Khandelwal, Richard E Galgon, Marwan Ali, Aaron M Joffe

**Affiliations:** 1 University of Washington, Harborview Medical Center, 325 Ninth Avenue, Box 359724, Seattle, WA 98104, USA; 2 University of Wisconsin School of Medicine and Public Health, Madison, WI, USA; 3 Northeast Ohio Medical University, School of Medicine, Rootstown, OH, USA

**Keywords:** Intubation, Videolaryngoscopy, Direct laryngoscopy, In-hospital cardiac arrest

## Abstract

**Background:**

Placement of advanced airways has been associated with worsened neurologic outcome in survivors of out-of-hospital cardiac arrest. These findings have been attributed to factors such as inexperienced operators, prolonged intubation times and other airway related complications. As an initial step to examine outcomes of advanced airway placement during in-hospital cardiac arrest (IHCA), where immediate assistance and experienced operators are continuously available, we examined whether cardiopulmonary resuscitation efforts affect intubation difficulty. Additionally, we examined whether or not the use of videolaryngoscopy increases the odds of first attempt intubation success compared with traditional direct laryngoscopy.

**Methods:**

The study setting is a large urban university-affiliated teaching hospital where experienced airway managers are available to perform emergent intubation for any indication in any out-of-the-operating room location 24 hours a day, 7 days-a-week, 365 days-a-year. Intubations occurring in all adults >18 years-of-age who required emergent tracheal intubation outside of the operating room between January 1, 2008 and December 31, 2012 were examined retrospectively. Multivariate logistic regression was used to estimate the odds of difficult intubation during IHCA compared to other emergent non-IHCA indications with adjustment for *a priori* defined potential confounders (body mass index, operator experience, use of videolaryngoscopy versus direct laryngoscopy, and age).

**Results:**

In adjusted analyses, the odds of difficult intubation were higher when taking place during IHCA (OR=2.63; 95% CI 1.1-6.3, p=0.03) compared to other emergent indications. Use of video versus direct laryngoscopy for initial intubation attempts during IHCA, however, did not improve the odds of success (adjusted OR = 0.71; 95% CI 0.35-1.43, p = 0.33).

**Conclusions:**

Difficult intubation is more likely when intubation takes place during IHCA compared to other emergent indications, even when experienced operators are available. Under these conditions, direct laryngoscopy (versus videolaryngoscopy) remains a reasonable first choice intubation technique.

## Background

Airway management in general, and placement of advanced airways in particular, during cardiac arrest is contentious. The “ABC” (i.e., airway, breathing, and circulation) method of remembering the correct protocol for cardiopulmonary resuscitation (CPR) was initially described more than 50 years ago [1]. The American Heart Association (AHA) adopted the mnemonic as the ABC system of CPR training in 1973 [2]. Based largely on data suggesting that interruptions in chest compressions worsen the chance of neurologically intact survival, the 2010 AHA guidelines on CPR and emergency cardiac care de-emphasized the primacy of airway maintenance and artificial respiration to shift the focus on maximizing uninterrupted circulation [3]. Indeed, the largest study of patients suffering out-of-hospital cardiac arrest (OHCA) to date reported that CPR with an advanced airway (either endotracheal intubation or supraglottic airway placement) was a significant predictor of poor neurological outcome [4]. Several factors have been proposed to explain the link between pre-hospital advanced airway management and worse outcomes, including operator experience and suboptimal execution of CPR procedures [5]. Based on data from simulation-based studies, preferential use of videolaryngoscopy (VL) over direct laryngoscopy (DL) during arrest situations has been suggested to minimize the effect of operator experience on intubation success. Improved laryngeal views, fewer intubation attempts, and shorter intubation times have been reported [6-9]. This would presumably result in fewer interruptions in CPR. These findings, however, have not been reported in situations where experienced operators are immediately available and high quality CPR is routine, such as may be the case during in-hospital cardiac arrest (IHCA). Thus, the aims of our study were to (1) describe whether IHCA itself is associated with difficult intubation compared to emergent non-IHCA intubations and (2) examine whether or not the use of VL during IHCA increases the odds of first attempt intubation success compared with traditional DL.

## Methods

### Study setting

The study site was Harborview Medical Center (HMC), a 413-bed urban medical centre in Seattle, WA, affiliated with the University of Washington. HMC is the only Level 1 trauma centre in a five state area (Washington, Wyoming, Alaska, Montana and Idaho) with 88 adult intensive care unit (ICU) beds with separate ICUs for medical, cardiac, trauma/surgical, burn, and neurology/neurosurgery patients. The institution’s out-of-operating room airway management model for all locations other than the Emergency Department (ED) (ward, ICU, remote locations such as radiology suites, etc.), is anaesthesiology-based, which consists of a paging system with notification to a pre-assigned anaesthesia airway team composed of an anaesthesia trainee or nurse anaesthetist and an attending anaesthesiologist. In the ED, primary intubation responsibility is shared between board-certified Emergency Medicine (EM) trained physicians and their trainees and the anaesthesiology service depending on the type of admission (medical, surgical, or trauma) with the anaesthesia-based airway team available 24 hours, 7 days per week as back-up. In both cases, a two-person airway management team is mandated. Additionally, members of the Department of Respiratory Therapy and bedside nurses familiar with emergency procedures attend all intubations. On the ward or in other remote locations, such as radiology or the outpatient clinic areas, dedicated “rapid response” nurses also attend and assist with intubations as part of a dedicated rapid response team. When intubations occur in the ED or ICU, the unit bedside nurses attend and assist the intubation as well. All VL intubations are performed using one of 3 available versions of the GlideScope® videolaryngoscopy system (i.e., the GVL, Ranger, or AVL Single Use).

### Study design

The Institutional Review Board of the University of Washington (Seattle, WA, USA) approved this study with a waiver of informed consent. This study was a retrospective, comparative study of all out-of-operating room intubations performed between January 1, 2008 and December 31, 2012 and contained in a prospectively collected out-of-operating room airway database. All patients who suffered an IHCA were identified using hospital “code blue” committee records. “Code blue” is the institution’s designation for cardiopulmonary arrest. This was crosschecked with the airway database to avoid misclassification of patients in whom cardiac arrest was misdiagnosed on the intubation note in the airway database; for example, situations in which a “code blue” response was called to summon rapid assistance for a deteriorating patient who was not in full cardiopulmonary arrest. In these instances, the responding providers who performed the intubation may have listed cardiac arrest as the underlying, albeit erroneous, diagnosis.

### Eligibility criteria

All patients ages 18 and older who were emergently intubated outside of the operating room (OR) were eligible. Only patients intubated during resuscitative efforts for IHCA comprised the IHCA group. Patients for whom a “code blue” response was called, but were not in full arrest, suffered the arrest in the peri-intubation period, or had an *in situ* tracheal tube when the arrest occurred were excluded. However, these patients remained in the pool of patients from which the non-IHCA group was selected.

### Data collection

All providers, regardless of departmental affiliation, complete the same intubation procedure note, which is contained within the electronic medical record (EMR). Intubation details, including (1) whether or not intubation occurred during IHCA, (2) the providers present and their experience level, (3) the initial intubation technique (DL or VL) used, (4) the laryngeal views attained, (5) the number of intubation attempts performed, and (6) any complications, were abstracted from the procedure notes. Demographic variables, including age, gender, height, and weight, were additionally retrieved from the EMR. All data were abstracted by one of the co-authors (MA) with independent verification on a random sample by the senior author (AMJ). In order to provide an appropriate control group and avoid systematic and sampling biases, patients emergently intubated for reasons other than IHCA were randomly selected from the airway database using the following described procedure. First, all cases from the airway database were imported into an Excel spread sheet (Microsoft Office Professional Edition 2003, Microsoft Corporation, Seattle, WA) and ordered in a descending fashion by date of service. Second, after excluding any entries outside the 5-year study period, any duplicate entries, any involving patients 18 years or younger (i.e., paediatric patients), and those involving IHCA, random numbers were generated and assigned to each case using the ‘RANDBETWEEN’ function. Control cases were then selected consecutively following the random number order. In the case of missing control case data, the deficient record was deleted and the next control record was selected for inclusion until two control cases were obtained for every IHCA case.

### Study endpoints and study definitions

The primary endpoints of the study were the odds of encountering a difficult intubation (DI) during IHCA and first attempt intubation success using VL versus DL during IHCA. DI was defined as an intubation requiring ≥3 attempts, >10 minutes to accomplish, or the need for a surgical airway. DI itself, pulmonary aspiration, and oesophageal intubation were considered airway complications. Operators were classified as either junior or senior. Definitions of senior operator vary widely in the literature, from “…completed at least 6 months of an anaesthesia residency or…a critical care medicine attending physician” [10] to “anesthesiologists and intensivists with experience in intubation procedures >5 years and experience in ICU >1 year” where “an operator was defined as an anesthesiologist if he had a formal anesthetic training of more than 24 months” [11]. For our study, we chose to use a somewhat conservative definition for senior operator. A junior operator was defined as anyone with <1 year of formal airway training. EM and anaesthesiology attending physicians, EM residents in their final year of training (having completed at least 2 years of EM training), anaesthesiology residents past their first clinical anaesthesia year (having completed an additional 4–6 weeks of formal airway training in their intern year), anaesthesiology fellows, and certified nurse anaesthetists were all considered senior operators. Fellows based in other departments, such as medicine or surgery, were classified on a case-by-case with consideration of their prior airway training. In the event of multiple intubation attempts, the experience of the operator was recorded as the one who performed the initial intubation attempt.

### Data analysis

Baseline demographic and clinical variables were compared between patients intubated during IHCA and those intubated emergently outside the OR for other non-IHCA indications using a two-sample Student’s t-test with assumption of unequal variances (Satterthwaite’s degrees of freedom) for continuous variables and Chi-square tests for categorical variables. Multivariate logistic regression was used to estimate the odds of DI during IHCA compared to other emergent non-IHCA indications. Models were adjusted for the following *a priori* defined potential confounders: BMI, operator experience and use of VL versus DL. In addition to these *a priori* defined potential confounders, age was included as a covariate as there was a statistically significant difference in age between the two groups in univariate analyses. Traditional external airway exam features, such as thyromental distance, mouth opening, Mallampati score, cervical spine mobility, and neck circumference were not included as potential confounders for two reasons. First, airway exams are not typically performed and/or documented for IHCA intubations. Second, and perhaps more importantly, even under controlled conditions, such as elective surgical cases, the external airway exam has severely limited predictive value [12]. The odds of first attempt intubation success per intubation technique (VL vs. DL) was similarly estimated using multivariate logistic regression models, again adjusted for *a priori* defined confounders (BMI, operator experience, and intubation during IHCA or not) and age. A two-sided α < 0.05 was considered statistically significant. Statistical analyses were performed using STATA statistical software, version 12.0 (StataCorp. College Station, TX).

## Results

Ten of the original 280 (3.5%) selected control cases had missing data and were replaced according to the randomization procedure. None of the IHCA-group had missing data. Demographic and intubation data for the entire group, stratified by exposure to IHCA, are presented in Table 1. Patients intubated during IHCA were older (61 years old ±16 vs. 56 ± 15, p < 0.01), experienced more difficult intubations (10% vs. 4%, p = 0.01), and suffered more pulmonary aspiration (4% vs. 0.5%, p < 0.01). The adjusted odds of encountering a DI during IHCA was 2.63 (95% CI 1.1-6.3, p = 0.03).

**Table 1:** Comparison of out-of-operating room intubations occurring during in-hospital cardiac arrest (IHCA) versus other emergent indications

	**All patients N = 420**	**IHCA N = 140**	**Non-IHCA N = 280**	**P-value**^**a**^
Age in years, mean (SD)	58 (16)	61 (16)	56 (15)	<.01
Male, n (%)	293 (70)	102 (73)	191 (68)	.30
BMI kg.m^−2^, median (IQR)	27 (23–33)	28 (24–34)	27 (23–33)	.43
Initial intubation technique, n (%)				<.01
Direct laryngoscopy	371 (88)	133 (95)	238 (85)	
Glidescope	49 (12)	7 (5)	42 (15)	
Initial glottic view, n (%)				.37
Grade 1	291 (70)	91 (65)	200 (71)	
Grade 2	97 (23)	35 (25)	62 (22)	
Grade 3	25 (6)	10 (7)	15 (5)	
Grade 4	7 (2)	4 (3)	3 (1)	
Operator Experience, n (%)				.06
Junior	129 (31)	35 (25)	94 (34)	
Senior	291 (69)	105 (75)	186 (66)	
First attempt success, n (%)	336 (78)	103 (74)	223 (80)	.16
Complications, n (%)				
Difficult intubation^b^	25 (6)	14 (10)	11 (4)	.01
Aspiration	7 (2)	6 (4)	1 (0.5)	<.01
Oesophageal intubation	5 (1)	0	5 (2)	.11

SD: standard deviation; BMI: body mass index; IQR: interquartile range.

^a^Two sample t-test with assumption of unequal variance, Chi-square test or Wilcoxon rank sum test.

^b^Difficult intubation is defined as ≥3 intubation attempts, >10 minutes in duration, or need for a surgical airway.

Demographic and intubation data for the entire group, stratified by initial intubation technique, are presented in Table 2. The adjusted odds of first attempt intubation success based on use of VL or DL were similar; OR = 0.71 (95% CI 0.35-1.43, p = 0.33).

**Table 2:** Comparison of all intubations based on initial airway technique

	**All patients N = 420**	**DL N = 371**	**VL N = 49**	**P-value**^**a**^
Age in years, mean (SD)	58 (16)	58 (16)	56 (16)	0.58
Male, n (%)	293 (70)	265 (71)	28 (57)	0.04
BMI in kg.m^−2^, median (IQR)	27 (23–33)	27 (23–33)	30 (23–35)	0.19
Initial glottic view, n (%)				0.06
Grade 1	291 (69)	257 (69)	34 (69)	
Grade 2	97 (23)	90 (24)	7 (14)	
Grade 3	25 (6)	19 (5)	6 (12)	
Grade 4	7 (2)	5 (1)	2 (4)	
Operator Experience, n (%)				0.32
Junior	129 (31)	117 (32)	12 (24)	
Senior	291 (69)	254 (68)	37 (76)	
First attempt success, n (%)	326 (78)	291 (78)	35 (71)	0.27
Complications, n (%)				
Difficult intubation^b^	25 (6)	21 (6)	4 (8)	0.49
Aspiration	7 (2)	7 (2)	0 (0)	0.33
Oesophageal intubation	5 (1)	4 (1)	1 (2)	0.56

DL: direct laryngoscopy; VL: videolaryngoscopy; SD: standard deviation; BMI: body mass index; IQR: interquartile range.

^a^Two sample t-test with assumption of unequal variance, Chi-square test or Wilcoxon rank sum test.

^b^Difficult intubation is defined as ≥3 intubation attempts, >10 minutes in duration, or need for a surgical airway.

## Discussion

In this study, we found that the odds of encountering a difficult intubation during IHCA was higher than during emergent intubations performed for other non-IHCA indications, even when factors such as operator experience were considered. Additionally, using VL for initial intubation attempts during IHCA, where experienced operators were available and involved, was not associated with increased odds of intubation success compared to traditional DL.

Compared to routine intubations performed in the operating room, higher rates of DI and airway related complications have been well documented in the out-of-operating room intubation literature. Rates of DI, hypoxemia (SpO_2_ < 90%), severe hypoxemia (SpO_2_ < 70%), oesophageal intubation, aspiration, and cardiac arrest reportedly occur in up to 12%, 22%, 26%, 7.4%, 5.9%, and 1.6% of patients, respectively [10,13-18]. Of particular relevance to our report, a number of groups have reported that intubation using DL is adversely affected during on-going chest compressions in simulation-based models [6,7,19-22]. However, association between CPR and performance of tracheal intubation in actual patients under IHCA conditions, where experienced operators are available and involved, has not been previously described. Thus, our study is the first to address this issue.

Our results are strengthened by the inclusion of only patients who were emergently intubated outside the controlled setting of the OR and controlling for operator experience. Still, even after adjustment for operator experience, the odds of DI during IHCA was over 2.5 times that for other emergent non-IHCA indications. This finding in isolation would support the preferential use of advanced airway technologies, such as VL devices to improve first attempt intubation success, and presumably, decrease the chances of intubation-related complications. In fact, a number of investigators have documented improvements in a variety of intubation related endpoints with the use of VL compared to DL in both human [23-31] and mannequin studies [32-35]. However, our results do not support this assertion. Differences between prior published studies and our study may account for this observation. For example, pre-hospital and mannequin based studies of intubation during CPR often include less experienced operators. Senior level operators performed nearly 70% of intubations in our study. In addition, the out-of-operating room intubation model at our institution is robust in that it mandates the presence of two operators. Thus, even when an inexperienced operator, such as a junior trainee actually performed the intubation, a more senior operator was present to provide assistance or take over if needed. The presence of a second operator has been previously reported to decrease DI and airway-related complications in out-of-operating room intubations [16]. Additionally, one cannot stress enough the importance of additional staff that is not available in the pre-hospital setting and not incorporated into simulation-based studies, such as respiratory therapists and bedside nurses, attending intubations. Lastly, it is worth noting that despite having similar initial laryngeal views during intubations for IHCA and non-IHCA, intubations were still more difficult during IHCA. Tracheal intubation is a multi-step dynamic process, which includes laryngeal sighting, delivery of the tube to the glottic opening, and finally, advancing the tube beyond the target into the trachea [36]. Regardless of whether DL or a non-channelled VL device such as the GlideScope® is used, either of the 2 later steps could potentially be affected by motion artefact from external chest compressions or the ergonomically unfavourable position of the operator, patient, or both. For these reasons, initial glottic view may not necessarily correspond with ease of securing the airway.

We acknowledge our study is firstly limited by its retrospective design and may be affected by an undetected confounding factor that cannot be accounted for by its design. Second, while all intubations in the IHCA group took place during resuscitative efforts, we cannot verify if they took place during on-going chest compressions, at the time of a scheduled pause, or during a requested stoppage. Roughly half of the intubations included in our analysis took place prior to implementation of the new AHA 2010 guidelines, which went live in November of that year. Our institution went live in January of 2011. However, this would only serve to weaken the association between IHCA and DI, not strengthen it. Thus, the significant increase in adjusted odds of DI during IHCA may be an underestimation of the true effect. Third, as already mentioned, the in-hospital intubation model is dissimilar to many other care settings, which limits the generalizability of our conclusions to care models different from ours. Also, VL use was proportionally low during initial intubation attempts overall, and thus our study may lack sufficient power to detect relevant differences. Nonetheless, our findings are consistent with a recently published prospective randomized trial of DL versus the GlideScope® for intubation of trauma patients [37] where the study setting was an urban university-affiliated institution with an airway management model quite similar to ours. Among all patients, the median [IQR] duration of intubation was significantly longer in patients randomized to the GlideScope® compared with DL (56 [40–81] seconds versus 40 [24–68] seconds; p < 0.001) and first attempt intubation success was similar (80% for GlideScope® and 81% for DL; p = 0.46). Although selection bias cannot be excluded (patients who were presumed to benefit from VL and therefore had it initially rather than DL), the data suggest that some of the proposed advantages of VL over DL are negated by experience and or/experienced supervision.

Finally, we would like to stress that our study did not evaluate the effects of different intubation techniques (i.e., VL vs. DL) on the actual performance of high quality CPR or on any contribution of airway management technique to return of spontaneous circulation.

## Conclusion

DI is more likely when intubation takes place during IHCA compared to other emergent conditions, and this should be taken into consideration when conceptualizing initial and subsequent intubation plans. Having experienced operators perform intubation at the outset or provide supervision to less experienced operators appears to mitigate some of the proposed advantages of VL over DL. Further prospective study of airway management during IHCA is warranted with specific attention to timing, duration, and its effects on high-quality CPR. Additionally, and importantly, any affect of advanced airway placement on patient-centred outcomes, such as return of spontaneous circulation and neurologic function at discharge for survivors, remains to be determined.

## Competing interests

The authors declare that they have no competing interests.

## Authors’ contributions

NK carried out statistical analysis, interpreted data, and co-authored the manuscript. REG helped interpret data and co-authored the manuscript. MA collected the data and co-authored the manuscript. AMJ conceived of the study, aided in data collection, interpreted data, and co-authored the manuscript. All authors read and approve the final manuscript.

## Pre-publication history

The pre-publication history for this paper can be accessed here:

http://www.biomedcentral.com/1471-2253/14/38/prepub

## References

[B1] Safar P Brown TC Holtey WJ Wilder RJ Ventilation and circulation with closed-chest cardiac massage in man JAMA 1961 176 574 576 10.1001/jama.1961.03040200010003 13745343

[B2] Mitka M Peter J Safar MD “Father of CPR” innovator, teacher, humanist JAMA 2003 289 2485 2486 12759308 10.1001/jama.289.19.2485

[B3] Morrison LJ Deakin CD Morley PT Callaway CW Kerber RE Kronick SL Lavonas EJ Link MS Neumar RW Otto CW Parr M Shuster M Sunde K Peberdy MA Tang W Hoek TL Böttiger BW Drajer S Lim SH Nolan JP Advanced Life Support Chapter Collaborators Part 8: Advanced life support: 2010 International Consensus on Cardiopulmonary Resuscitation and Emergency Cardiovascular Care Science with Treatment Recommendations Circulation 2010 122 S345 S421 10.1161/CIRCULATIONAHA.110.971051 20956256

[B4] Hasegawa K Hiraide A Chang Y Brown DF Association of prehospital advanced airway management with neurologic outcome and survival in patients with out-of-hospital cardiac arrest JAMA 2013 309 257 266 10.1001/jama.2012.187612 23321764

[B5] Wang HE Yealy DM Out-of-hospital endotracheal intubation: where are we? Ann Emerg Med 2006 47 532 541 10.1016/j.annemergmed.2006.01.016 16713780

[B6] Han SK Shin DH Choi PC Utility of the Pentax-AWS without interruption of chest compression: comparison of the Macintosh laryngoscope with the Pentax-AWS in manikin model Resuscitation 2010 81 69 73 10.1016/j.resuscitation.2009.09.031 19919888

[B7] Shin DH Choi PC Han SK Tracheal intubation during chest compressions using Pentax-AWS®, GlideScope®, and Macintosh laryngoscope: a randomized crossover trial using a mannequin Can J Anaesth 2011 58 733 739 10.1007/s12630-011-9524-4 21630116

[B8] Kim YM Kim JH Kang HG Chung HS Yim HW Jeong SH Tracheal intubation using Macintosh and 2 video laryngoscopes with and without chest compressions Am J Emerg Med 2011 29 682 686 10.1016/j.ajem.2010.02.014 20825887

[B9] Kim YM Kang HG Kim JH Chung HS Yim HW Jeong SH Direct versus video laryngoscopic intubation by novice prehospital intubators with and without chest compressions: a pilot manikin study Prehosp Emerg Care 2011 15 98 103 10.3109/10903127.2010.514087 21034232

[B10] Schwartz DE Matthay MA Cohen NH Death and other complications of emergency airway management in critically ill adults. A prospective investigation of 297 tracheal intubations Anesthesiology 1995 82 367 376 10.1097/00000542-199502000-00007 7856895

[B11] De Jong A Molinari N Terzi N Mongardon N Arnal JM Guitton C Allaouchiche B Paugam-Burtz C Constantin JM Lefrant JY Leone M Papazian L Asehnoune K Maziers N Azoulay E Pradel G Jung B Jaber S AzuRéa Network for the Frida-Réa Study Group Early identification of patients at risk for difficult intubation in the intensive care unit: development and validation of the MACOCHA score in a multicenter cohort study Am J Respir Crit Care Med 2013 187 832 839 10.1164/rccm.201210-1851OC 23348979

[B12] Yentis SM Predicting difficult intubation – worthwhile exercise or pointless ritual? Anaesthesia 2002 57 105 109 10.1046/j.0003-2409.2001.02515.x 11871945

[B13] Mort TC Emergency tracheal intubation: complications associated with repeated laryngoscopic attempts Anesth Analg 2004 99 607 613 15271750 10.1213/01.ANE.0000122825.04923.15

[B14] Griesdale DE Bosma TL Kurth T Isac G Chittock DR Complications of endotracheal intubation in the critically ill Intensive Care Med 2008 34 1835 1842 10.1007/s00134-008-1205-6 18604519

[B15] Martin LD Mhyre JM Shanks AM Tremper KK Kheterpal S 3,423 emergency tracheal intubations at a university hospital: airway outcomes and complications Anesthesiology 2011 114 42 48 10.1097/ALN.0b013e318201c415 21150574

[B16] Jaber S Amraoui J Lefrant JY Arich C Cohendy R Landreau L Calvet Y Capdevila X Mahamat A Eledjam JJ Clinical practice and risk factors for immediate complications of endotracheal intubation in the intensive care unit: a prospective, multiple-center study Crit Care Med 2006 34 2355 2361 10.1097/01.CCM.0000233879.58720.87 16850003

[B17] Woodall N Frerk C Cook TM Can we make airway management (even) safer? – lessons from national audit Anaesthesia 2011 66 Suppl 2 27 33 22074076 10.1111/j.1365-2044.2011.06931.x

[B18] Simpson GD Ross MJ McKeown DW Ray DC Tracheal intubation in the critically ill: a multi-centre national study of practice and complications Br J Anaesth 2012 108 792 799 22315326 10.1093/bja/aer504

[B19] Tandon N McCarthy M Forehand B Carlson JN Comparison of intubation modalities in a simulated cardiac arrest with uninterrupted chest compressions Emerg Med J 2013 epub ahead of print 10.1136/emermed-2013-202783 23850885

[B20] Komasawa N Ueki R Kohama H Nishi S Kaminoh Y Comparison of pentax-AWS airwscope video laryngoscope, airtraq optic laryngoscope, and Macintosh laryngoscope during cardiopulmonary resuscitation under cervical stabilization: a manikin study J Anesth 2011 25 898 903 10.1007/s00540-011-1218-0 21901329

[B21] Xanthos T Stroumpoulis K Bassiakou E Koudouna E Pantazopoulos I Mazarakis A Demestiha T Iacovidou N Glidescope® videolaryngoscope improves intubation success rate in cardiac arrest scenarios without chest compressions interruption: a randomized cross-over manikin study Resuscitation 2011 82 464 467 10.1016/j.resuscitation.2010.12.011 21272986

[B22] Maruyama K Tsukamoto S Ohno S Kobayashi K Nakagawa H Kitamura A Hayashida M Effect of cardiopulmonary resuscitation on intubation using a Macintosh laryngoscope, the AirWay scope, and the gum elastic bougie: a manikin study Resuscitation 2010 81 1014 1018 10.1016/j.resuscitation.2010.03.041 20605669

[B23] Ng I Sim XL Williams D Segal R A randomised controlled trial comparing the McGrath® videolaryngoscope with the straight blade laryngoscope when used in adult patients with potential difficult airways Anaesthesia 2011 66 709 714 10.1111/j.1365-2044.2011.06767.x 21564049

[B24] Ng I Hill AL Williams DL Lee K Segal R Randomized controlled trial comparing the McGrath videolaryngoscope with the C-MAC videolaryngoscope in intubating adult patients with potential difficult airways Br J Anaesth 2012 109 439 443 22677878 10.1093/bja/aes145

[B25] Jungbauer A Schumann M Brunkhorst V Börgers A Groeben H Expected difficult tracheal intubation: a prospective comparison of direct laryngoscopy and video laryngoscopy in 200 patients Br J Anaesth 2009 102 546 550 19233881 10.1093/bja/aep013

[B26] Stroumpoulis K Pagoulatou A Violari M Ikonomou I Kalantzi N Kastrinaki K Xanthos T Michaloliakou C Videolaryngoscopy in the management of the difficult airway: a comparison with the Macintosh blade Eur J Anaesth 2009 26 218 222 10.1097/EJA.0b013e32831c84d1 19237983

[B27] Kaplan MB Hagberg CA Ward DS Brambrink A Chhibber AK Heidegger T Lozada L Ovassapian A Parsons D Ramsay J Wilhelm W Zwissler B Gerig HJ Hofstetter C Karan S Kreisler N Pousman RM Thierbach A Wrobel M Berci G Comparison of direct and video-assisted views of the larynx during routine intubation J Clin Anesth 2006 18 357 362 10.1016/j.jclinane.2006.01.002 16905081

[B28] Marrel J Blanc C Frascarolo P Magnusson L Videolaryngoscopy improves intubation condition in morbidly obese patients Eur J Anaesth 2007 24 1045 1049 10.1017/S0265021507000889 17608975

[B29] Healy D Maties O Hovord D Kheterpal S A systematic review of the role of videolaryngoscopy in successful orotracheal intubation BMC Anaesthesiol 2012 12 32 10.1186/1471-2253-12-32 PMC3562270 23241277

[B30] Sun DA Warriner CB Parsons DG Klein R Umedaly HS Moult M The GlideScope video laryngoscope: randomized clinical trial in 200 patients Br J Anaesth 2005 94 381 384 15567809 10.1093/bja/aei041

[B31] Lim Y Yeo SW A comparison of the GlideScope with the Macintosh laryngoscope for tracheal intubation in patients with simulated difficult airway Anaesth Intensive Care 2005 33 243 247 15960409 10.1177/0310057X0503300215

[B32] Savoldelli GL Schiffer E Abegg C Baeriswyl V Clergue F Waeber JL Comparison of the Glidescope®, the McGrath®, the Airtraq® and the Macintosh laryngoscopes in simulated difficult airways Anaesthesia 2008 63 1358 1364 10.1111/j.1365-2044.2008.05653.x 19032306

[B33] Ray DC Billington C Kearns PK Kirkbride R Mackintosh K Reeve CS Robinson N Stewart CJ Trudeau T A comparison of McGrath and Macintosh laryngoscopes in novice users: a manikin study Anaesthesia 2009 64 1207 1210 10.1111/j.1365-2044.2009.06061.x 19825056

[B34] Healy DW Picton P Morris M Turner C Comparison of the glidescope, CMAC, storz DCI with the Macintosh laryngoscope during simulated difficult laryngoscopy: a manikin study BMC Anesthesiol 2012 12 11 10.1186/1471-2253-12-11 22720884 PMC3519500

[B35] McElwain J Malik MA Harte BH Flynn NM Laffey JG Comparison of the C-MAC® laryngoscope with the Macintosh, Glidescope®, and Airtraq® laryngoscopes in easy and difficult laryngoscopy scenarios in manikins Anaesthesia 2010 65 483 489 10.1111/j.1365-2044.2010.06307.x 20337620

[B36] Levitan RM Heitz JW Sweeney M Cooper RM The complexities of tracheal intubation with direct laryngoscopy and alternative intubation devices Ann Emerg Med 2011 57 240 247 10.1016/j.annemergmed.2010.05.035 20674088

[B37] Yeatts DJ Dutton RP Hu PF Chang YW Brown CH Chen H Grissom TE Kufera JA Scalea TM Effect of video laryngoscopy on trauma patient survival: a randomized controlled trial J Trauma Acute Care Surg 2013 75 212 219 10.1097/TA.0b013e318293103d 23823612

